# Dengue Virus Type 2: Protein Binding and Active Replication in Human Central Nervous System Cells

**DOI:** 10.1155/2013/904067

**Published:** 2013-11-04

**Authors:** Ma Isabel Salazar, Marissa Pérez-García, Marisol Terreros-Tinoco, María Eugenia Castro-Mussot, Jaime Diegopérez-Ramírez, Alma Griselda Ramírez-Reyes, Penélope Aguilera, Leticia Cedillo-Barrón, María Martha García-Flores

**Affiliations:** ^1^Laboratorio de Inmunología Celular e Inmunopatogénesis, Departamento de Inmunología, Escuela Nacional de Ciencias Biológicas, Instituto Politécnico Nacional, Prolongación Manuel M. Carpio y Plan de Ayala S/N, Colonia Santo Tomás, 11340 México, DF, Mexico; ^2^Servicio de Neurocirugía, Unidad Médica de Alta Especialidad, Centro Médico Nacional Siglo XXI, Avenida Cuauhtemoc No. 330, Colonia Doctores, 06720 México, DF, Mexico; ^3^Laboratorio de Patología Cerebrovascular, Instituto Nacional de Neurología y Neurocirugía “Manuel Velasco Suárez”, Avenida Insurgentes Sur No. 3877, Colonia La Fama, 14269 México, DF, Mexico; ^4^Departamento de Biomedicina Molecular, Centro de Investigación y de Estudios Avanzados, Instituto Politécnico Nacional, Avenida Instituto Politécnico Nacional No. 2508, Colonia Zacatenco, 07360 México, DF, Mexico; ^5^Unidad de Investigación Médica en Inmunología, Hospital de Pediatría, Centro Médico Nacional Siglo XXI, Avenida Cuauhtemoc No. 330, Colonia Doctores, 06720 México, DF, Mexico

## Abstract

An increased number of dengue cases with neurological complications have been reported in recent years. The lack of reliable animal models for dengue has hindered studies on dengue virus (DENV) pathogenesis and cellular tropism *in vivo*. We further investigate the tropism of DENV for the human central nervous system (CNS), characterizing DENV interactions with cell surface proteins in human CNS cells by virus overlay protein binding assays (VOPBA) and coimmunoprecipitations. In VOPBA, three membrane proteins (60, 70, and 130 kDa) from the gray matter bound the entire virus particle, whereas only a 70 kDa protein bound in white matter. The coimmunoprecipitation assays revealed three proteins from gray matter consistently binding virus particles, one clearly distinguishable protein (*~*32 kDa) and two less apparent proteins (100 and 130 kDa). Monoclonal anti-NS3 targeted the virus protein in primary cell cultures of human CNS treated with DENV-2, which also stained positive for NeuH, a neuron-specific marker. Thus, our results indicate (1) that DENV-2 exhibited a direct tropism for human neurons and (2) that human neurons sustain an active DENV replication as was demonstrated by the presence of the NS3 viral antigen in primary cultures of these cells treated with DENV-2.

## 1. Introduction

In recent years, several neurological disorders, such as delirium, coma, amnesia, seizures, meningitis, encephalitis, encephalomyelitis, Guillian-Barré syndrome, neuropathies, and intracranial hemorrhages, have been shown to be associated with dengue infections [1–6]. Factors secondary to dengue infection caused by thrombocytopenia leading to hemorrhages, liver failure, and electrolyte imbalances are some determinants of encephalopathy during the illness, not of encephalitis. It is crucial to differentiate both conditions that usually occur during dengue hemorrhagic fever and dengue shock syndrome [6].

Virus antigen has been detected in the central nervous system (CNS) in dengue patients whether the outcomes are fatal [7] or not fatal [8–10]. In endemic areas, the evidence of dengue cases with neurological involvement has increased [11–13]. 

A recent study of fatal dengue cases established that CNS involvement occurs more frequently than was previously reported; dengue virus (DENV) was found in 48.8% of the cerebrospinal fluids (CSF) analyzed. The neurologic diagnoses for these patients were the following: encephalitis (46.3%), meningoencephalitis (34.1%), and meningitis (19.5%) [14]. These authors reported that the major clinical manifestations observed in these individuals were fever, headache, irritability, breathlessness, vomiting, muscle pain, tiredness, abdominal pain, somnolence, restlessness, dizziness, cough, seizure, coma, and neck stiffness [14]. 

The neurological complications in dengue may be due to different conditions occurring in severe disease; these conditions have been grouped into metabolic disturbances, autoimmune reactions, and direct viral invasion [6]. In the CSF of some dengue patients, the virus NS1 protein [15], specific IgM antibodies [5, 12, 16], or virus particles [7, 12] have been found. Some reports indicate that antibodies in the CSF originate from intrathecal synthesis in the CNS [17]. The four DENV serotypes have been associated with neurological complications in dengue cases [18–21].

DENV classically has been considered a nonneurotropic virus; however, the accumulating evidence for DENV neurotropism [11, 14, 22] in an important proportion of cases suggests direct viral encephalitis. This would involve tropism and direct neuronal infiltration by DENV to the human CNS, where proteins functioning as receptor and cells sustaining virus replication can be found. In this work, to demonstrate the presence of proteins in human brain tissues, which could interact with DENV, we used VOPBA, coimmunoprecipitation, and immunofluorescence assays to establish DENV-2 binding to CNS proteins and active replication in primary cell cultures. In these assays, proteins from Vero cells and brain tissue from neonatal mice (laboratory models well known for susceptibility to DENV infection) [23, 24] were used as controls. We identified membrane proteins in human brain tissue potentially involved in the binding and internalization processes as well as active virus replication taking place in neurons.

## 2. Material and Methods

### 2.1. Human Brain Tissues

As part of some surgical procedure in epileptic patient, brain tissue is removed from the temporal lobe and in some other pathologies other regions such as motor cortex. This tissue is used primarily for histopatologic and laboratory studies, and sometimes the fresh tissue can be recovered for investigation. Authorization for this study was obtained from the Ethics Committee of the Pediatric Hospital, CMNSXXI, IMSS; informed consent was obtained from all patients and from the parents of those patients who were underage (≤18 years old).

In this study, we used fragments (remnants) of brain tissue that had been surgically removed from four patients, two females (9 and 15 years old) and two males (8 and 10 years of age). Each one of these tissues was processed surgically by a neurosurgeon to separate the gray matter (corresponding to cellular bodies and dendrites) and white matter (including axons), which were processed separately.

### 2.2. Separation of Cell Membrane Proteins

To obtain cell membrane proteins from cell cultures used as controls (Vero and MLg) as well as brain tissues (human or mouse), the FractionPrep Cell Fractionation Kit (Biovision, Inc. Salt Lake City, UT, USA) was used, following the manufacturer's instructions. The system provides reproducible extraction of cytosol, nucleus, membrane/particulate, and cytoskeletal fractions from cell samples, in this case, it was used to obtain the membrane/particulate fraction only.

Briefly, tissue (human brain tissue from gray or white matter and neonatal mouse brain tissue) was cut into little pieces with a razor, disrupted manually with a pestle, and finally homogenized in 4 mL of cold PBS by using a glass Dounce homogenizer. Each suspension was transferred to an individual conical tube (15 mL) and centrifuged (500 ×g; 5 min) to collect the corresponding cells. For preparation, the cytosolic fraction was discarded; each pellet was then mixed with cold membrane extraction buffer-A (400 *μ*L). After, each sample was homogenized by stirring (15 sec), membrane extraction buffer-B (22 *μ*L) was added. Each sample was vortexed (5 sec), incubated on ice (1 min), remixed (4 sec), and then centrifuged (1000 ×g; 5 min). Each supernatant was immediately transferred to its corresponding clean, prechilled tube, and 10 *μ*L aliquot of each sample was obtained, and then the rest of the sample was kept as the membrane/particulate fraction at −20°C until further use in VOPBA. In each aliquot, the proteins were quantified in a Nanodrop 2000 UV/Vis spectrophotometer (Thermo Scientific, Reedwood City, CA, USA).

### 2.3. Virus Overlay Protein Binding Assays (VOPBA)

To establish which proteins in the white and gray matter from human CNS were involved in virus binding, VOPBA were carried out. Briefly, for each preparation, Vero and MLg cells, neonatal mice brain tissue, and human white and gray matter of membrane proteins (80 *μ*g) were subjected to SDS-12% PAGE electrophoresis. The protein band profile was then verified in Coomassie stained gels. Following electrophoresis, the proteins were electrotransferred to nitrocellulose membranes (253 mAmp; 2 h). The membranes were blocked with 5% low-fat milk in PBS (4°C; overnight) and then washed three times with 0.15% Tween 20 in PBS (10 min). The membranes were incubated at room temperature for 2 h with 2 × 10^6^ PFU of either DENV-2 strain New Guinea [25] or DENV-2 strain Yuc18500 [26] suspended in 2% low-fat milk then rinsed with 0.15% Tween 20-PBS (three times; 10 min each at room temperature). Each membrane was then incubated (4 h at room temperature) with monoclonal anti-dengue 3H5.1 antibody (Millipore, Darmstadt, Germany) diluted 1 : 100 in PBS, followed by rinsing with PBS—0.15% Tween 20 (three times; 10 min each at room temperature). Subsequently, a 1 : 1000 dilution of the secondary antibody, anti-mouse IgG-HRP (Invitrogen, Frederick, MD, USA), in PBS was incubated with the membrane (2 h at room temperature), followed by three washes of 10 min with PBS—0.15% Tween 20—and a final 10 min rinse with PBS. To identify the recognized proteins bands, the reaction was revealed by using ECL western blotting detection chemiluminescence system (Amersham Biosciences, Freiburg, Germany) and radiographic film (Kodak, Windsor, CO, USA).

### 2.4. Coimmunoprecipitation Assays

Due to the results obtained in VOPBA, where three protein bands were recognized by DENV-2 in gray matter and only one in white matter, only portions corresponding to gray matter were used for the coimmunoprecipitation assays. Supernatants from C6/36 monolayers infected with a MOI = 0.1 of either DENV-2 strain New Guinea or DENV-2 strain Yuc18500 [26] were collected six days after infection. After allowing the supernatants to interact overnight with 8% polyethylene glycol, the virus from these supernatants were concentrated by centrifuge (10,000 ×g; 30 min at 4°C). The pellets were then resuspended in PBS-Complete (Roche, Mannheim, Germany); protein concentrations were determined; and aliquots were stored at −20°C until use. For protein interactions, the adequate volume of CNS gray matter proteins and the virus particles were resuspended in 1 mL of 1 x RIPA buffer (20 mM Tris-HCl, 150 mM NaCl, 1 mM EGTA, 1% NP-40, 1% sodium desoxycolate with complete protease inhibitor cocktail).

For coimmunoprecipitation, the protein interaction was carried out as described in 1x RIPA buffer (4°C overnight) with constant rocking, by using proteins from CNS gray matter (600 *μ*g) without virus (control) or mixed with DENV-2 virus protein (150 *μ*g). Thereafter, 10 *μ*L of 3H5.1 anti-dengue antibody and 10 *μ*L of 4G2 antibody (Millipore, Darmstadt, Germany) were added to each tube and incubated in a laboratory rocker (3 h; 4°C). Then, protein G/A agarose (20 *μ*L; Santa Cruz, CA, USA) was added to the mixture (2 h; 4°C). The tubes were centrifuged (12 000 ×g; 20 min), the supernatants were discarded, and the pellets were rinsed extensively with PBS-Complete (5 times; 1 mL). Finally, the pellet was resuspended in PBS-Complete (50 *μ*L), 6x Laemmli buffer containing 5% ß-mercaptoethanol (10 *μ*L), placed in a boiling water bath for 7 min, and loaded into the wells of 12% SDS-PAGE for electrophoresis. The gel was stained with Coomassie, destained (40% methanol, 10% acetic acid), and then examined to determine the proteins from CNS gray matter that had coimmunoprecipitated with virus E protein.

### 2.5. Establishment of CNS Primary Cell Cultures

The initial standardization for the cell culture and antibody markers was performed in a brain tissue extracted from newborn BALB/c mice (<5 days after birth) (see Supplementary Figure 1 available online at http://dx.doi.org/10.1155/2013/904067). Later, the conditions were also tested for the human brain tissue and optimized accordingly. For human tissue, an expert neurologist removed the affected tissue and harvested the small remnants of healthy tissue under aseptic conditions; these were placed in tubes with DMEM containing 10% FBS and immediately transferred to the lab for processing. Sterile conditions were used throughout the procedures.

Tissue (~40 mm^3^) was washed several times with sterile PBS and cut into small pieces (3–5 mm), which were transferred to Eppendorf tubes and digested (15 min; 37°C) with papain (300 *μ*L; ≥10 U/mg protein; Sigma-Aldrich Co). The pieces were rinsed twice with DMEM-10% FBS (500 *μ*L) and fresh medium was added (1000 *μ*L); then, the tissue pieces were mechanically disaggregated by using a Pasteur pipette, and the resulting mixture was filtered by using a sterile mesh (100 *μ*m). The resulting cell suspension was collected and 100 *μ*L was transferred to each of the eight wells, pretreated with Matrigel (Becton-Dickinson, Bedford, MA, USA), in a Lab-Tek II chamber slide system (Nunc, Rochester, NY, USA). Then DMEM with 10% FBS (250 *μ*L) was added to each well.

After incubation (24 h), to favor neurons growth the medium was replaced with a 1 : 1 DMEM-F12 medium (Life Technologies, Foster City, CA, USA) mixture (250 *μ*L) supplemented with 10% FBS. Cultures were transferred to 37°C in a 5% CO_2_ atmosphere and were maintained up to two weeks in this Matrigel-pretreated Labtek chambers. The cultures were treated with DENV-2 on day 2 and were examined 3–5 days after the infection. Because the number of viable cells in wells varied, we used a MOI = ~5. The virus stock was resuspended in serum-free DMEM-F12 medium (300 *μ*L); for infection, 75 *μ*L of the suspension was inoculated into each well and allowed to interact with the cells (45 min; 37°C; 5% CO_2_-atmosphere). For the mock-infected control, the corresponding wells were treated with serum-free DMEM-F12 (75 *μ*L). Then, fresh DMEM-F12-10% FBS (300 *μ*L) was added and the cells incubated (37°C; 5% CO_2_-atmosphere). The cultures were examined each day by using a bright-field microscope, until at day 5 after infection the medium was carefully removed and the cells were rinsed with PBS. The cells were then fixed in 1 : 1 methanol : acetone (300 *μ*L; 10 min; −20°C); the mixture was removed; and wells were rinsed with PBS before proceeding with the immunofluorescence staining.

### 2.6. Immunofluorescence Assays to Identify NeuH Marker and NS3 Viral Protein

The dilution used for the anti-NS3 monoclonal antibody was standardized in C6/36 cell monolayers infected for seven days (supplementary Figure 2). Monoclonal antibodies against the NS3 protein have been used to establish dengue tropisms in humans and in a mouse model [27]. Here, we used a monoclonal antibody raised and tested at the Centro de Investigación y Estudios Avanzados (CINVESTAV) [28]. The dilution used for the rabbit antibody against human Neu H (Millipore, Darmstadt, Germany) was established during the standardization of primary brain cells cultures. The anti-NS3 monoclonal antibody was diluted 1 : 100 in 300 *μ*L of PBS and the rabbit anti-Neu H antibody, 1 : 50 also in 300 *μ*L of PBS. All the staining procedures were done in a humidity chamber to prevent desiccation. The brain tissue cell cultures were blocked with 3% BSA in PBS (40 min; room temperature). Thereafter, the blocking solution was removed and the cells were rinsed with PBS—0.02% Tween 20; the cell cultures were incubated with the anti-Neu H antibody (150 *μ*L; 1 h; room temperature), rinsed, and incubated with the anti-NS3 antibody under the same conditions. Between and after the addition of antibodies, three 5-min rinses with PBS were performed. The secondary antibodies (diluted 1 : 100 in 150 *μ*L) were allowed to interact (1 h; in the dark) with the preparations in each well. The secondary antibodies used were an goat anti-mouse IgG labeled with FITC (Life Technologies, Foster City, CA, USA) and an goat anti-rabbit IgG labeled with rhodamine (Invitrogen, Frederick, MD, USA). Between and after the addition of antibodies, three 5-min, rinses with PBS were performed. Each chamber was removed from the Lab-Tek slide and was mounted by using Vectashield (Vector Laboratories Inc, Burlingame, CA, USA) and observed by using an epifluorescence (Nikon) or confocal microscope (Olympus).

## 3. Results

### 3.1. Three Proteins in CNS Gray Matter Bound DENV-2 Particles

**Figure 1:**
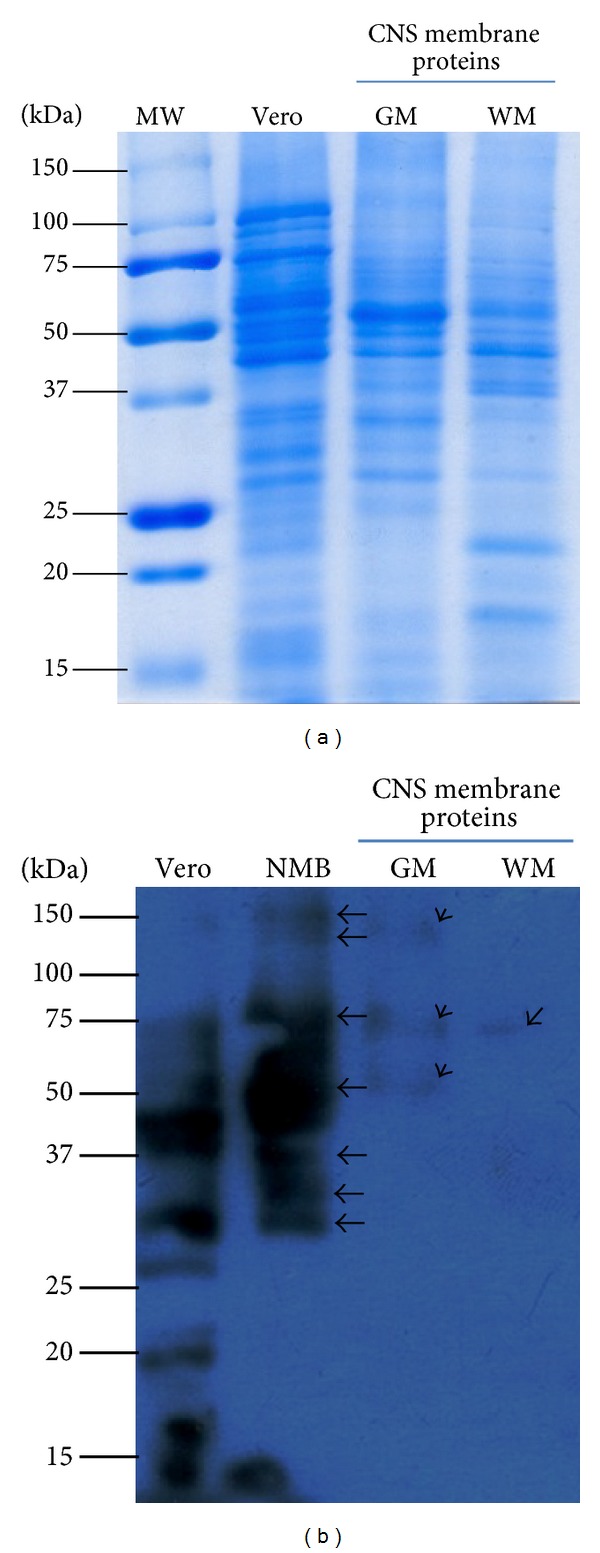
Binding of dengue virus type 2 (DENV-2) to membrane proteins of the central nervous system (CNS). To establish which proteins in the human CNS were involved in binding DENV, VOPBA were carried out by using the entire DENV particles precipitated with polyethylene glycol and membrane proteins obtained from either white or gray matter from human tissue. Three membrane proteins (60, 70, and 130 kDa) from gray matter were found to bind the entire viral particle, while only one of them (70 kDa) was so identified in the white matter. MW: molecular weight; Vero: membrane proteins from Vero cells; NMB: membrane proteins form neonatal mouse brain; GM: membrane proteins from gray matter; WM: membrane proteins from white matter; kDa: kilodaltons. Arrows point to the proteins detected to bind virus particles in this assay. (b); (a) shows proteins in SDS-PAGE 12%.

To identify interactions between proteins located on the surface of human CNS cells and DENV-2 particles, we carried out VOPBA. For these assays, we used remnants of specimens of normal human brain tissue taken at biopsy to obtain only membrane proteins through cell fractioning. Then, the patterns and integrity of membrane proteins were verified (Figure 1(a)).

Membrane proteins from Vero cells and brain cells from neonatal mice were used as controls for the binding assays. In Vero cells, a greater number of proteins were revealed to bind virus particles in comparison with human brain tissue, whereas seven proteins were shown to attach in neonatal brain tissue proteins (Figure 1(b)), with approximate molecular weights of such brain proteins of 32, 35, 40, 65, 80, 140, and 170 kDa. Membrane proteins obtained from CNS gray matter, which corresponds to cellular bodies and dendrites, exhibited three distinct proteins that bound to DENV particles in VOPBA assays, with molecular weights approximately of 60, 70, and 130 kDa, while only one membrane protein (of approximately 70 kDa) from CNS white matter (enclosing axons) bound DENV particles.

### 3.2. Two Proteins in CNS Gray Matter Interacted with DENV Envelope (E) Protein

**Figure 2:**
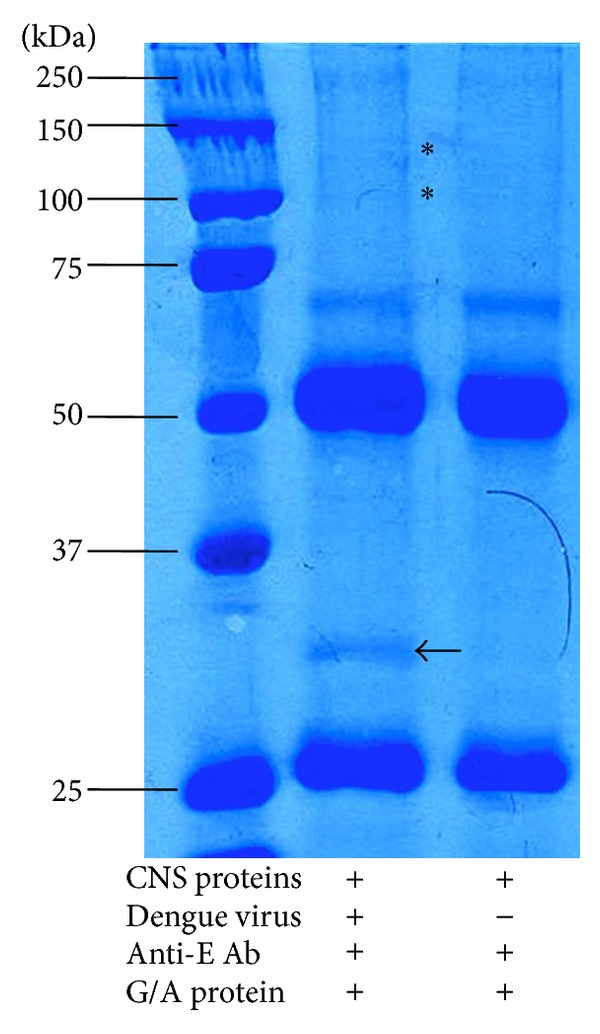
Coimmunoprecipitation of central nervous system (CNS) proteins with dengue E viral protein. Coimmunoprecipitation assays revealed that four CNS proteins consistently bound with dengue virus particles. One major protein with molecular weight of 32 kDa and other two minor proteins with molecular weights of 100 and 130 kDa were obtained. These were not present in the control with no virus. Black arrows point to CNS protein bands that bound dengue virus E protein. *Protein bands with a minor signal.

In VOPBA, the protein interactions with the virus particles occurred under the reduced conditions that are required for the SDS-PAGE; therefore, to detect native proteins in CNS gray matter lysates interacting with E protein, we used coimmunoprecipitation assays. In the coimmunoprecipitation assays, we identified one prominent protein (approximately 32 kDa) as well as two less noticeable proteins (approximately 100 and 130 kDa) that interact with DENV-2 E protein release from virus particles. None of these bands appeared in the control reaction and consistent results were obtained in two independent experiments (Figure 2). In both lanes for problem and control reactions, protein bands (25 and 50 kDa), which corresponded to the light and heavy chains of the antibody used in these reactions, were observed, as was a 60 kDa band corresponding to virus E protein.

### 3.3. DENV-2 Replicated in Neurons as Indicated by Presence of Nonstructural Protein, NS3

**Figure 3:**
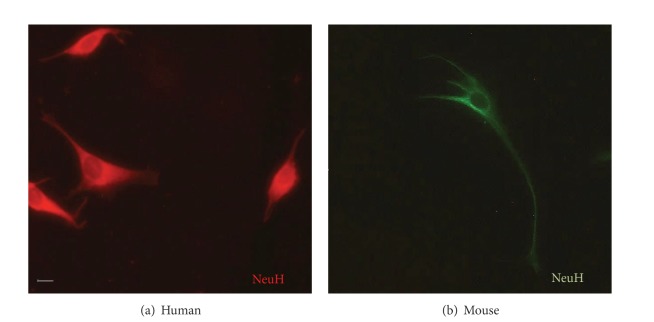
Neurons detected by using anti-NeuH in immunofluorescence assays. In immunofluorescence assays, neurons in primary cell cultures of either human central nervous system (CNS) (a) or (b) mouse brain tissue were identified with the neuron-specific cellular marker, NeuH. The secondary antibodies used contained rhodamine (a) or FITC (b) as fluorochromes. In green, FITC signal in mouse neurons is shown and in red rhodamine signal is observed in human neurons. Original magnification was 400x, but the pictures were cropped to improve presentation.

In immunofluorescence assays, we observed abundant neurons in cultures (Figure 3); however, in some of these primary cultures, some astrocytes were also distinguished (Supplementary Figure 1). Morphology between these two different cells was very distinctive: neurons appeared as smaller, fusiform cells, whereas astrocytes were bigger and star-shaped, exhibiting gross prolongations.

**Figure 4:**
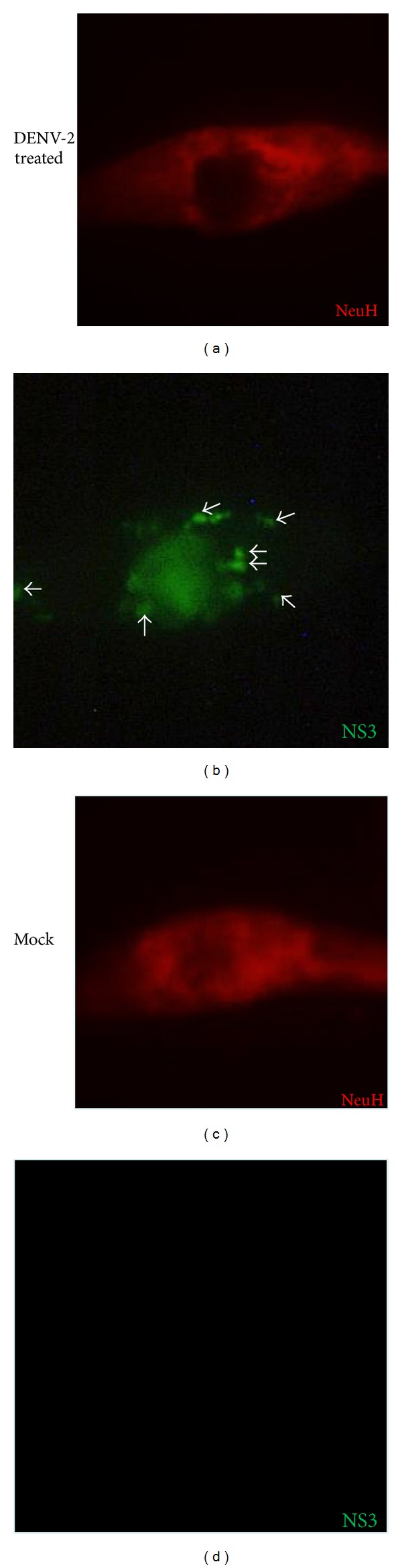
Detection of DENV replication in neurons. Cells in mixed primary cell cultures from human CNS treated with DENV-2 were examined after 5 days by indirect immunofluorescence in an epifluorescence microscope. The monoclonal antibody raised against NS3 DENV allowed the assessing of the active viral replication in the brain primary cell cultures, (b) and (d) while the antibody cell markers enable the identification of the cell type as neurons. (c) and (d) Original magnification was 600x, but the pictures were cropped to improve presentation. Arrows point to virus replication sites in human neurons.

**Figure 5:**
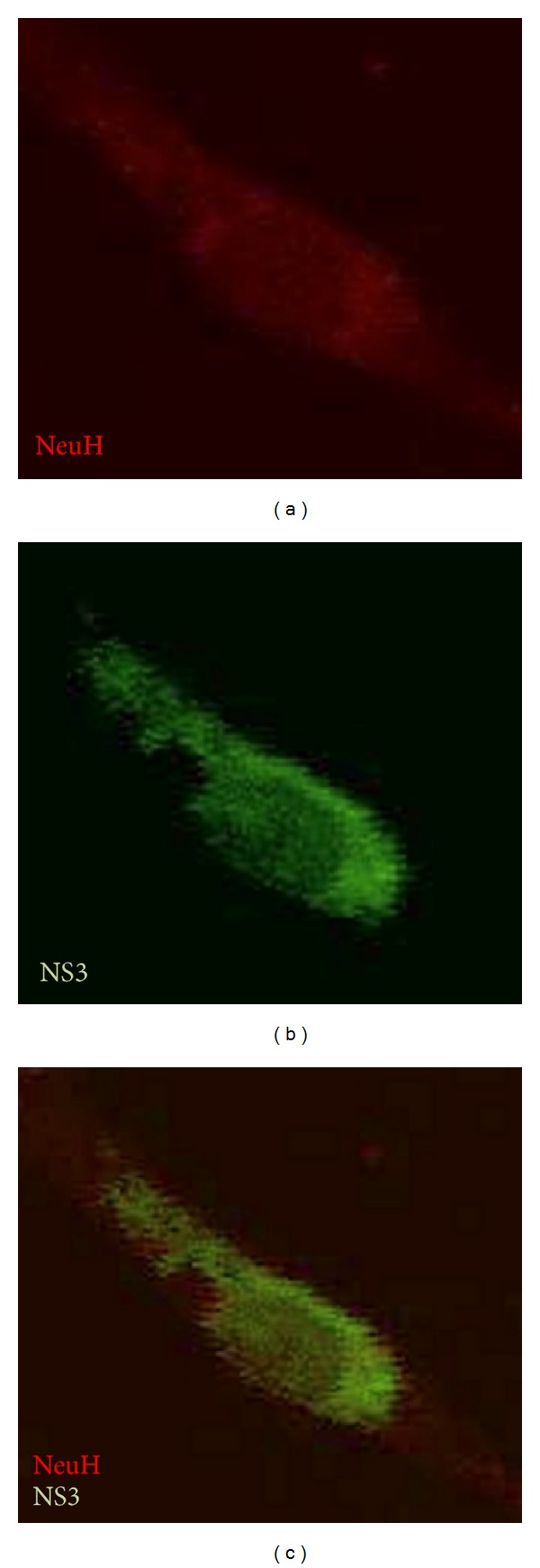
DENV-2 NS3 protein expresses in neurons. Detection of the viral replication by indirect immunofluorescence in primary cultures of human neural cells treated with DENV type 2 using an anti-NS3 monoclonal antibody by confocal microscopy. (b) and (c) Original magnification was 600x, but the pictures were cropped to improve presentation.

A monoclonal antibody raised against NS3 DENV allowed us to assess the active viral replication in primary brain cell cultures, while the antibody cell markers enabled the identification of the cell type. When primary brain cell cultures, which had been exposed to DENV-2, were stained with anti-NS3 monoclonal antibody approximately 40% of the neurons in culture exhibited NS3 expression. This protein was clearly distinguishable in the perinuclear area, as well as in defined areas in the cellular body in some cells and more profusely distributed in others (Figure 4 and data not shown). NS3-positive cells were also positive for the Neu H marker clearly indicating their neuronal origin (Figure 5).

## 4. Discussion

Among the clinical variety of dengue manifestations, some neurological syndromes were initially reported to occur in 1–25% of the cases [29]. However, the frequency of such reports has increased in recent years [13, 14, 22, 30]. The origin of such complications is unclear as is the mechanism by which DENV reaches the brain during infection. Profound damage to the endothelium occurs during dengue episodes, this is mediated by self-reacting antibodies [31], by cytokines such as TNF-*α* [32–34] and IFN-*γ* [35], by other biochemical substances as nitric oxide [36], by apoptotic processes [37], and by the plausible cell destruction mediated by the complement cascade or cytotoxic events. One of these events, or a combination thereof, may alter the blood-brain barrier and contribute to facilitating entrance of the virus into the brain tissue. However, to date, direct tropism of DENV for human brain cells has not been established.

Here, we demonstrated that proteins present chiefly in the gray matter of the human CNS could bind either intact DENV particles or the viral E protein. Gray matter includes neuronal cell bodies and dendrites. Thus, these data point to the presence of neuronal proteins that are able to bind DENV particles or the E protein alone. Moreover, we demonstrated the ongoing replication process in primary cell cultures by using a monoclonal antibody against NS3 viral protein. Taken together, these data suggest that DENV may exhibit a direct tropism for human neural cells *in vivo*. With the new detection techniques, accumulating evidence indicates that it is probable that DENV causes encephalitis more often than was initially thought [4, 14, 38, 39].

An important question is how DENV overcomes the blood-brain barrier. Although in the capillary endothelium in the blood-brain barrier the cells possess characteristic and restrictive thigh juctions, this region is often overcome in some normal physiological processes [40]. In fact, leukocytes can transverse the endothelium through a transcellular route [41]. Thus, the blood-brain barrier is transversed under certain conditions, not only by T and B lymphocytes [42, 43] but also by monocytes that regularly reach the perivascular spaces [41, 44, 45]. It is clear that microglia promotes the recruitment of activated monocytes in the brain in response to TNF-*α* upregulation in mice with inflammatory liver injury [46].

In dengue patients, elevated TNF-*α* levels, which seems to correlate with disease severity, are observed [47, 48]. Also, monocytes are one of the principal cellular types infected by DENV *in vivo* [49]. Moreover, liver involvement and injury are common clinical findings in dengue patients [50, 51]. Thus, DENV could reach brain tissue due the pathophysiological conditions taking place during dengue infections.

Virus tropism is mediated by the cellular conditions that enable infection and replication, including the presence of receptors and coreceptors that allow virus entry. The existence of candidate neuronal receptors for DENV has been examined *in vitro *[52, 53]. Early studies suggested that a 65 kDa membrane protein, which is present in mouse brain cells and the human neuroblast cell line SK-N-SH, may be responsible for susceptibility to DENV infection [53]. In the present study, the approximately 60 kDa protein identified in membranes from neonatal mouse brain and from human gray matter may correspond to this previously reported protein [53].

Interestingly, a 70 kDa protein corresponding to a heat shock protein (HSP) has been previously reported [54], we identified a protein of that same molecular weight binding DENV particles in gray and white matter and in MLg fibroblastic cell line (data not shown). Additionally, a 130 kDa membrane protein of human hepatocytes (HEpG2 cell line) was shown to bind DENV particles of serotypes 3 and 4, but not 2 by VOPBA [55].

For the VOPBA, the membrane proteins were processed (reduced with ß-mercaptoethanol) and electrophoresed in SDS-PAGE; thus no native proteins were involved in the interaction with the virus particle. On the other hand, in coimmunoprecipitation assays, the initial interactions occur in their native state for both the cell membrane and the virus particle proteins. Later, the E protein and those that interacted with it precipitated with an anti-E protein specific polyclonal antibody and protein A/G agarose (Santa Cruz, CA) and were examined in SDS-PAGE. Thus, the proteins identified by this second method have a particular relevance.

In these coimmunoprecipitation assays, we identified a 32 kDa protein that bound to DENV-2 E protein; a DENV-2-binding protein of the same size human monocytes and T and B lymphocytes has been reported [56, 57]. Only the 130 kDa protein was identified by both assays. The discrepancies noted in the proteins identified by these two methods may result from different types of virus-cell-protein interactions. We have tried to identify these proteins by microsequencing; however, our results showed that this corresponded to an unnamed protein product, so they have not yet been conclusive.

There are reports of the presence of RNA of positive polarity in the CNS [39]; however this finding does not necessarily indicate ongoing replication. In contrast, presence of the replicative intermediate is indeed indicative of viral replication in the cell. Also there is a report of preferential involvement of gray matter in myelitis cases associated to dengue [58]. Here, we showed evidence of viral replication in neurons by detecting the NS3 protein, a protein that is present in the cell only after the viral RNA has been translated, the polyprotein cut, and the replication sites assembled. In primary cell cultures, we identified the ongoing replication in neurons from the motor cortex in human CNS. Further studies are necessary to establish the identity of the receptor/coreceptor molecules and the virus molecular mechanism to reach the brain and neurons in the human host.

## 5. Conclusions

DENV-2 exhibited a direct tropism for human neurons, which are permissive to infection and sustain an active DENV replication, as was demonstrated by the presence of the NS3 viral antigen in primary cultures of these cells. Some of the proteins identified herein may act as receptors or co-receptors during CNS infection with DENV.

## Supplementary Material

Supplementary Figure 1: Cellular markers for neurons and astrocytes. Two cellular types were identified in our primary cell cultures using specific antibodies, Neu H for neurons (B) and GFAP for astrocytes (D), secondary antibody was coupled to FITC. After staining the samples were process in an epifluorescence microscope. Arrows in bright field pictures point to cells in the primary cultures that were not stained with the tested marker. Original magnification was 600X, but the pictures were cropped to improve presentation.
Supplementary Figure 2: Detection of NS3 protein in infected C6/36 cells. To validate the specificity of the monoclonal antibody to detect dengue NS3 protein, DENV infected cultures were examined at different times post-infection and compared to mock-infected cultures of C6/36 cells. The figure shows monolayers of C6/36 cells after 7 days of infection with DENV and control. The signal is specific for the infected cell culture. Original magnification was 400X.
