# Supplementary figures and images for: Dengue Virus Type 2: Protein Binding and Active Replication in Human Central Nervous System Cells
Source: ScientificWorldJournal. 2013 Nov 4;2013:904067. doi: 10.1155/2013/904067 (PMC3835358; doi:10.1155/2013/904067)

## Slide 1
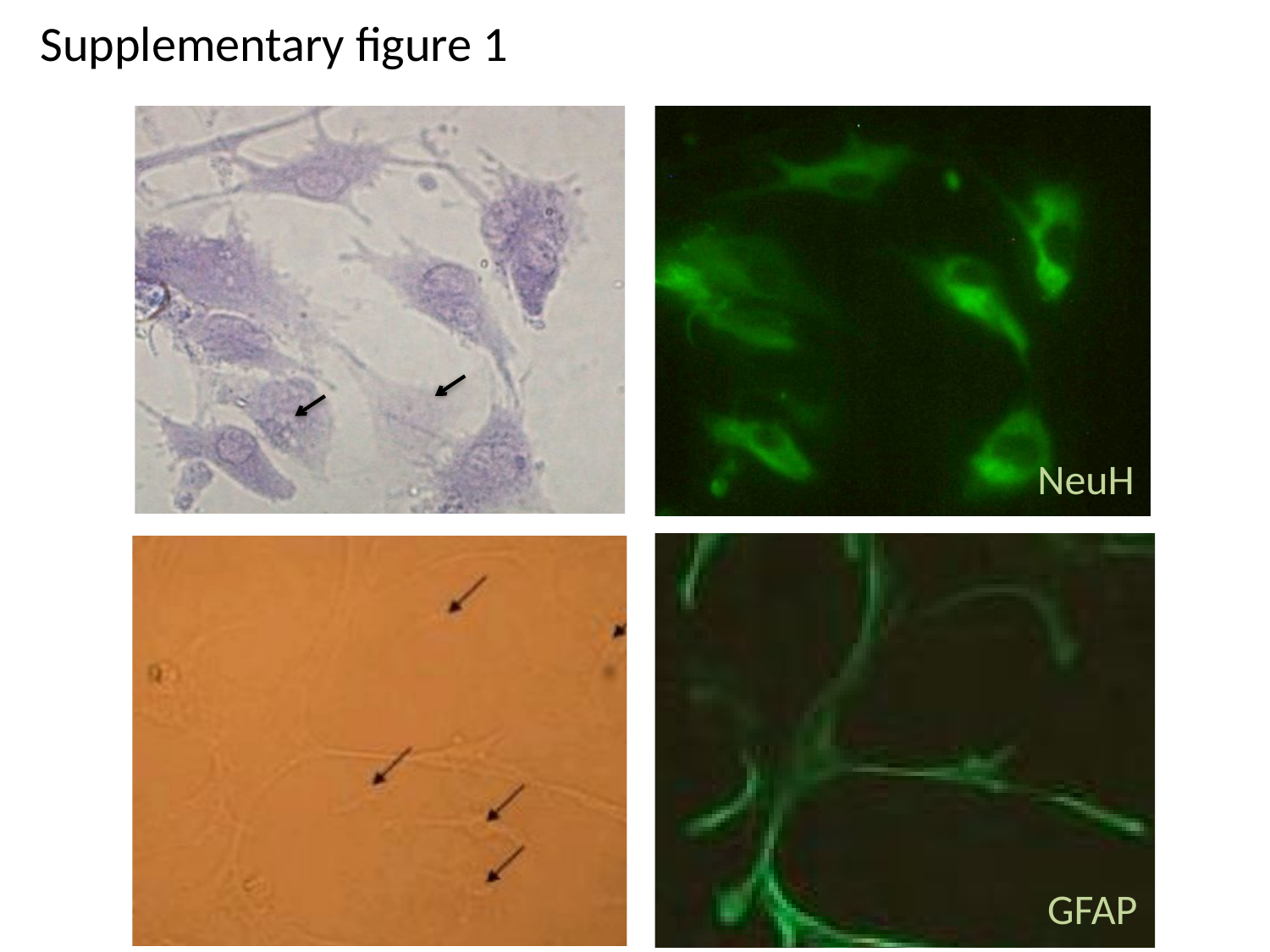

Supplementary figure 1
NeuH
GFAP
GFAP

Supplement: Supplementary file 1 — Supplementary Figure 1: Cellular markers for neurons and astrocytes. Two cellular types were identified in our primary cell cultures using specific antibodies, Neu H for neurons (B) and GFAP for astrocytes (D), secondary antibody was coupled to FITC. After staining the samples were process in an epifluorescence microscope. Arrows in bright field pictures point to cells in the primary cultures that were not stained with the tested marker. Original magnification was 600X, but the pictures were cropped to improve presentation. Supplementary Figure 2: Detection of NS3 protein in infected C6/36 cells. To validate the specificity of the monoclonal antibody to detect dengue NS3 protein, DENV infected cultures were examined at different times post-infection and compared to mock-infected cultures of C6/36 cells. The figure shows monolayers of C6/36 cells after 7 days of infection with DENV and control. The signal is specific for the infected cell culture. Original magnification was 400X. [file 904067.f1.pptx]

## Slide 1
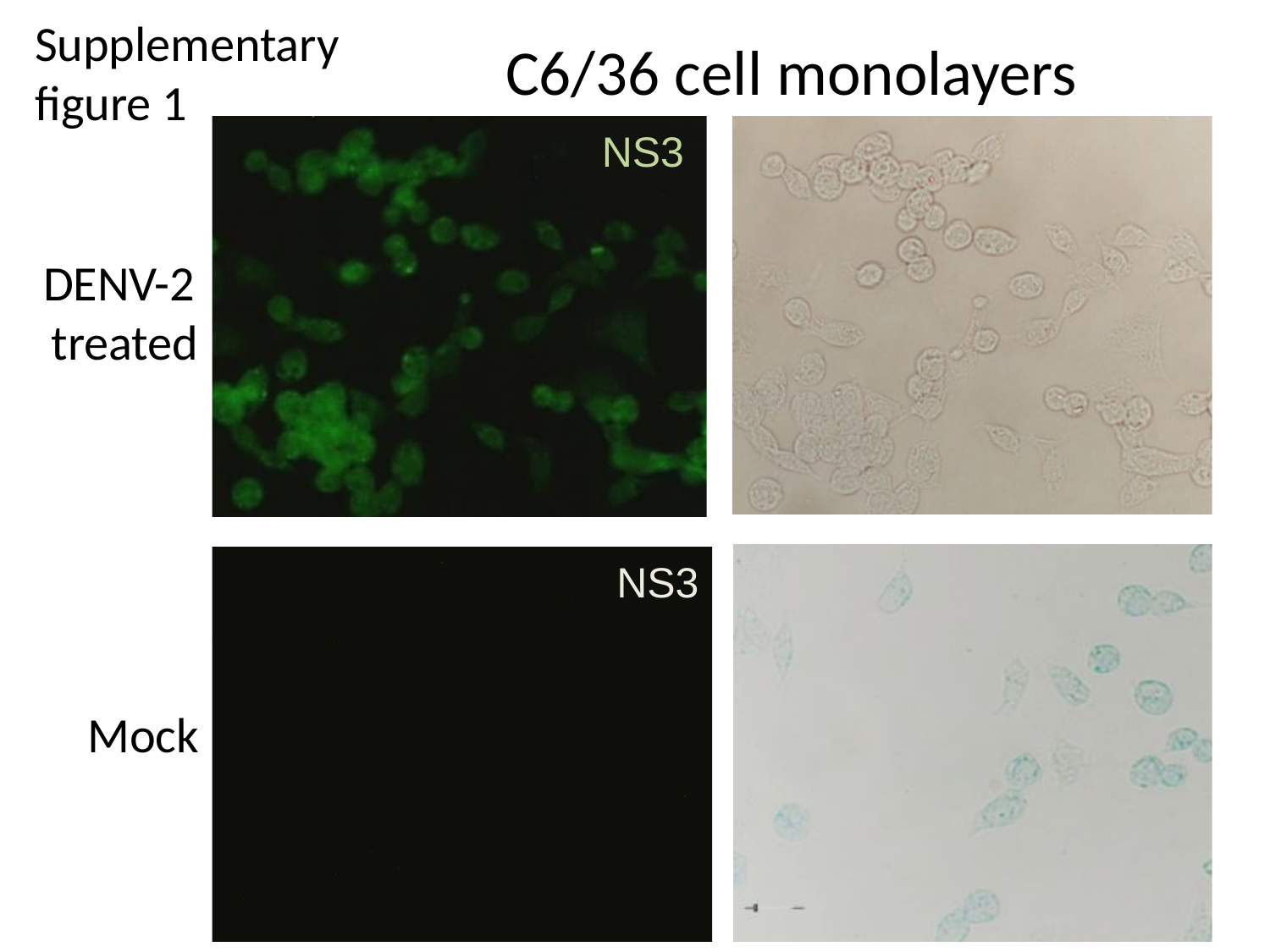

Supplementary
figure 1
C6/36 cell monolayers
NS3
DENV-2
treated
NS3
Mock

Supplement: Supplementary file 2 [file 904067.f2.pptx]
